# *When play turns perilous*: syncope in a young girl unmasked by electrocardiogram!

**DOI:** 10.1093/ehjcr/ytaf249

**Published:** 2025-05-26

**Authors:** Anagh T. Shetru, Kalyan Munde, Samkit Mutha

**Affiliations:** Department of Cardiology, Grant Government Medical College and Sir JJ Group of Hospitals, Mumbai 400008, India; Department of Cardiology, Grant Government Medical College and Sir JJ Group of Hospitals, Mumbai 400008, India; Department of Cardiology, Grant Government Medical College and Sir JJ Group of Hospitals, Mumbai 400008, India

A previously healthy 4-year-old girl experienced two sudden episodes of syncope while playing. Both episodes occurred abruptly, with no warning signs, and she regained consciousness spontaneously within seconds. There were no seizure-like movements, incontinence, or postictal confusion.

She was initially evaluated at a primary healthcare clinic, where she was diagnosed with seizures and started on antiepileptic medication. However, the absence of typical seizure features and the recurrence of syncope prompted further evaluation.

Her developmental history was normal, with no family history of epilepsy, sudden cardiac death, or inherited arrhythmias. Aside from a normal cardiovascular examination, her lab workup was unremarkable. What initially seemed like a seizure was something entirely different once we performed an electrocardiogram (ECG).

1. What is the most likely rhythm diagnosis based on this ECG?

- Second-degree Atrioventricular (AV) block (Mobitz I)
- Sinus bradycardia
- Complete heart block with prolonged QT interval
- Atrial fibrillation
- Junctional Bradycardia

Answer: C. Complete heart block with prolonged QT interval

## Discussion and explanation

Electrocardiogram shows AV dissociation and a slow, wide QRS rhythm, indicating ventricular escape, classic for complete heart block. Although QTc is visibly prolonged, we should keep in note that Bazett’s formula, QTc = QT/√RR, tends to overcorrect the QT interval at low rates, leading to an overestimation that can result in falsely prolonged QTc.

In a 4-year-old, normal QTc is <440 ms; here it exceeds 500 ms, suggestive of LQTS, which has a high risk of Torsades de Pointes and death.

Long QT syndrome (LQTS) with AV block is unusual, accounting for approximately 5% of cases and has a high mortality rate.^1^

2. Which of the following gene mutations is most commonly associated with both Long QT Syndrome and conduction system disease like complete heart block?

- KCNQ1
- KCNH2
- SCN5A
- CACNA1C
- MYH7

Answer: C. SCN5A

## Discussion and explanation

Long QT syndrome with complete AV block is rarely reported. Mutations in HERG (LQTS2), SCN5A (LQTS3), and SCN4B (LQTS10) are linked to this abnormality.^2^

SCN5A encodes the cardiac sodium channel (NaV1.5). Mutations lead to LQTS Type 3 and are associated with QT prolongation and conduction disorders, such as CHB and sick sinus syndrome.

In our case, genetic analysis revealed a heterozygous variant of unknown significance in Exon 41 of the FLNC gene. The FLNC gene encoding filamin C is primarily associated with myopathies and arrhythmogenic cardiomyopathies. The association of *FLNC* with channelopathies has been described by Neethling *et al*. in 2016, who reported an interaction between *FLNC* and *KCNE2* (potassium voltage-gated channel) known to be causative for LQTS.

**Figure:**
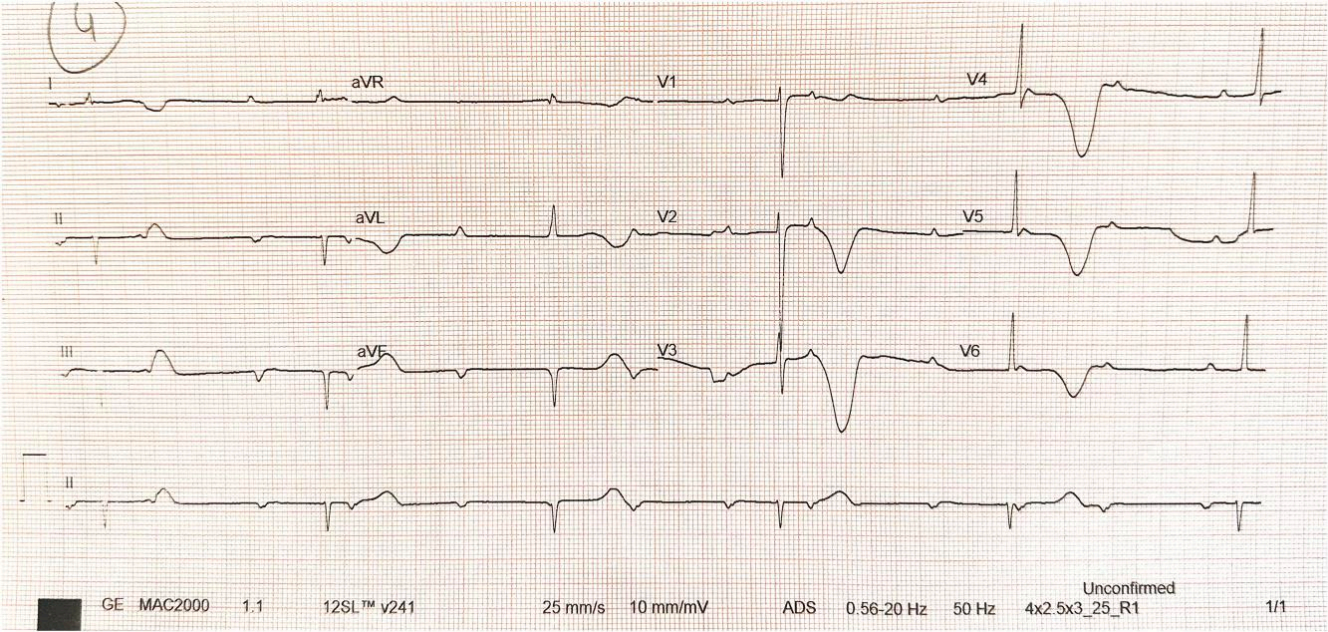


3. What is the next best step in management?

- Reassure and repeat ECG in 6 months
- Start IV lidocaine immediately
- Admit for permanent pacemaker implantation and start beta-blockers
- Beta-blockers alone
- Genetic testing before any intervention

Answer: C. Admit for permanent pacemaker implantation and start beta-blockers

## Discussion and explanation

This form of LQTS with AV block can manifest before birth or during neonatal life and has been associated with a guarded prognosis.^3^

β-Blockers are recommended as the first-line drug in all LQTS patients, except those with a very slow heart rate.^4^ Non-selective β-blockers nadolol and propranolol have better efficacy in inhibiting adrenergic activation to reduce the arrhythmic risk for inherited LQTS.

Our child was bradycardic due to CHB. Initiating beta-blockers without a pacemaker could worsen bradycardia. Therefore, permanent pacemaker implantation first, then cautious beta-blocker initiation, is the correct step. Genetic testing is important, but not an emergency step.

## Supplementary Material

ytaf249_Supplementary_Data

## Data Availability

The data underlying this article are available in the article and in its online Supplementary material.

## Supplementary material

**Consent:** The legal guardians of the patient have provided direct written consent for the inclusion of their child’s information in this manuscript.

**Funding:** This research received no specific grant from any funding agency in the public, commercial, or not-for-profit sectors.
