# Supplementary material for: When play turns perilous: syncope in a young girl unmasked by electrocardiogram!
Source: Eur Heart J Case Rep. 2025 May 26;9(6):ytaf249. doi: 10.1093/ehjcr/ytaf249 (PMC12143956; doi:10.1093/ehjcr/ytaf249)
Supplement: ytaf249_Supplementary_Data [file ytaf249_supplementary_data.zip › ytaf249_Supplementary_Data.zip]

**Supplementary Material: *When play turns perilous*: syncope in a young girl unmasked by electrocardiogram!**

- ECG after a pacemaker implantation.

- Atrial sensed ventricular-paced rhythm
- Qtc – 428 ms

Parameters LR- 80bpm, USR – 140bpm
